# Association Between Unstable Angina and CXCL17: a New Potential Biomarker

**DOI:** 10.1515/med-2019-0080

**Published:** 2019-12-08

**Authors:** Fu-han Gong, Xiao-qiang Xiao, Xue-ping Zhang, Li Long, Sheng Huang, Xue-sheng Wang, Zhen-lin Shu, Yong-sheng Yang

**Affiliations:** 1 Department of Cardiology, Tongren Municipal People’s Hospital, No.120 Taoyuan Avenue, Chuandong Education park, Bijiang District, Tongren City 554300, China; 2 Department of Clinical Laboratory, Tongren Municipal People’s Hospital, Tongren 554300, China; 3 Department of Ophthalmology, Tongren Municipal People’s Hospital, Tongren 554300, China

**Keywords:** CXCL17, angina pectoris, coronary angiography, Gensini score, Lipid metabolism indicators, high sensitivity C-reactive protein

## Abstract

Atherosclerosis and chemokines are strongly related, but the role of the chemokine CXCL17 in atherogenesis is still poorly understood. We aim to investigate the serum CXCL17 levels in different stages of patients with coronary heart disease and explore whether these differences contribute to atherosclerosis. In the current prospective study, we enrolled 48 patients with unstable angina (UA), 51 patients with stable angina (SA) and 41 patients for the control group (CG). All subjects were diagnosed by coronary angiography and Gensini score was used to evaluate the severity of coronary artery disease. The CXCL17 levels were determined using ELISA, while lipid metabolism indicators and high sensitivity C-reactive protein (hs-CRP) were detected by automatic biochemical analyzer. We observed that the unstable angina group had higher CXCL17 levels compared with the stable angina and the control group. The logistic regression analysis showed that CXCL17 was an independent risk factor for unstable angina. Our results showed that CXCL17 was also statistically correlated with hs-CRP, while it was irrelevant with Gensini score. CXCL17 levels were associated with activity of inflammatory response and the instability of atherosclerotic plaques. These results suggest that CXCL17 elevation may be a potential new biomarker of unstable angina.

## 1 Introduction

Cardiovascular diseases are the leading causes of death worldwide. It is well known that the common and important pathological process of most cardiovascular diseases is atherosclerosis. Mechanistically, the rupture of atherosclerotic plaque leads to thrombosis which clogs the lumen and subsequently results in ischemia of the distal blood supply area, which is recognized as the main cause of acute cardiovascular and cerebrovascular events. However, the pathogenesis of atherosclerosis has not yet been fully elucidated.

Low-grade chronic inflammations are the key characteristics and potential pathogenic factors in cardiometabolic diseases, especially atherosclerosis [1]. The activated immune and non-immune cells produce a set of cytokines, including type I IFNs, pro-inflammatory cytokines, and chemokines, which interact with their corresponding receptors to orchestrate the next phase of immune and inflammatory responses. Thus, excessive activation of these signals are deleterious to the homeostasis of the affected cardiovascular system [2]. Recent studies have found that GPR35, the receptor for chemokine (C-X-C motif) ligand 17 (CXCL17), is closely related to cardiovascular diseases [3, 4]. CXCL17 which is abnormally expressed in primary colon cancer and breast cancer plays an important role in chemotactic monocytes, macrophages and mature or immature dendritic cells [5, 6], and even is involved in the angiogenesis for tumor development and regulation of the expression of vascular endothelial growth factor [5, 6, 7]. However, the function of CXCL17 in the development of cardiovascular diseases, especially in atherosclerosis, is unclear. Therefore, the aim of this study is to investigate the relationship between CXCL17 and atherosclerosis-related diseases.

## 2 Patients and Methods

### 2.1 Subjects

For our study we enrolled patients of the Department of Cardiology of Tongren Municipal People’s Hospital, from January 2017-October 2017. A total of 140 subjects were included in our study: 48 patients with UA, 51 patients with SA and 41 patients for CG. The primary inclusion criterion was clinical chest pain. All patients completed coronary angiography within three days of admission. After coronary angiography, the patients diagnosed as coronary heart disease (CHD) were divided into unstable and stable angina, while the normal coronary arteries were included in the control group. CHD was defined as coronary stenosis ≥50% and Gensini score was calculated according to the results of coronary angiography. Considering that stress and acute inflammatory response may affect the changes of chemokines, our study excluded patients with acute myocardial infarction, acute or chronic inflammatory diseases such as infectious diseases, chronic congestive heart failure, neoplasms, autoimmune diseases and pregnant women. Percutaneous coronary intervention was performed in patients with ischemic symptoms and coronary stenosis ≥75%.

### 2.2 Ethics statement

This study was approved by the ethics committee of Tongren Municipal People’s Hospital and conducted according to the principles described in the Declaration of Helsinki. Written informed consent was obtained from all study participants.

### 2.3 Sample preparation

All subjects underwent 3 ml venous blood draw to test Troponin T (cTNT) and Brain natriuretic peptide (BNP) immediately at hospitalization. Another 3 ml venous blood sample used to test CXCL17 and biochemical indicators was collected in the morning after 8 hours of fasting. The samples were left at room temperature for 30 minutes to naturally solidify and successively centrifuged to extract the serum. The samples were then stored in -40 centigrade refrigerators and used for detecting the concentration of CXCL17 after three months.

### 2.4 Index assays

The concentration of CXCL17 was measured by ELISA kit (purchased from Bio-Swamp, NO:HM11279) and the procedure was performed according to the instructions. Kit detection range from 15pg/ml to 1200pg/ml, sensitivity: ≤3pg / ml. The indexes of blood lipid including total cholesterol, triglyceride, low density lipoprotein, high density lipoprotein and high sensitivity C-reactive protein (hs-CRP) were measured by an automatic biochemical analyzer.

### 2.5 Statistical Analysis

All statistical analyses were carried out with the SPSS 17.0 software and the data were presented as the mean ± SD. Categorical variables were presented as counts and percentages. Independent sample t-tests or ANOVA were performed to compare groups of continuous variables, and χ2 analysis was used to compare categorical data. Spearman correlation coefficients were calculated for the associations between the CXCL17 levels and the Gensini score or hs-CRP. Logistic regression was used to determine the relationship between CXCL17 and unstable angina after adjusting for confounding factors. All tests were two-sided, and significance was set at P<0.05.

## 3 Results

### 3.1 The variation of characteristics among CHD patients in different three groups

As shown in Table 1, the comparison among CHD patients with SA, UA and CG group showed statistically significant higher CXCL17 levels in the UA compared to the SA group (UA 461.11±85.42 vs SA 339.67±95.11, p<0.001). Interestingly, no significant difference was found between SA and CG (SA:339.67±95.11 vs CG:325.44±98.09, p=0.466). Troponin T (cTNT) and hs-PCR in the UA group was significantly higher than in the SA and CG. No differences in age, sex, BMI, EF, TIMI blood flow and the lipid parameters were found among the three groups, while the incidence of smoking, hypertension and diabetes were significantly higher in the UA group.

**Table 1:** Comparison of the characteristics between patients with SA, UA and control group.

	Control (n=41)	SA (n=51)	UA (n=48)	F/χ^2^	P (CT vs SA)	P (CT vs UA)	P (SA vs UA)
Age (years)	58.99±9.722	62.15±8.67	59.72±12.41	2.924	0.025	0.590	0.075
Sex (M/F)	23/18	37/14	35/13	5.307	0.100	0.097	0.967
BMI	24.63±3.47	23.86±1.46	24.79±2.36	1.953	0.144	0.763	0.066
Smokeing (cases)	18	27	32	6.692	0.389	0.031	0.164
Hypertension (cases)	8	31	18	13.814	< 0.001	0.063	0.021
Diabetes (cases)	1	11	9	7.655	0.007	0.015	0.727
TC (mmol/L)	4.03±1.09	3.89±1.25	4.25±1.15	1.125	0.588	0.383	0.138
HDL (mmol/L)	1.09±0.28	0.98±0.26	1.05±0.26	2.060	0.054	0.564	0.161
TG (mmol/L)	1.71±1.20	1.95±1.26	1.93±1.12	0.544	0.343	0.383	0.950
LDL (mmol/L)	2.63±0.80	2.55±1.02	2.73±0.85	0.483	0.653	0.627	0.328
Hs-CRP (g/L)	2.48±1.52	1.98±0.70	5.61±3.52	36.349	0.292	<0.001	<0.001
CXCL17 (pg/ml)	325.44±98.09	339.67±95.11	461.11±85.42	30.166	0.466	<0.001	<0.001
EF (%)	63.40±5.68	64.59±5.03	62.92±8.25	2.59	0.325	0.815	0.203
TIMI (grade 3 /<grade 3)	40/1	50/1	46/2	0.951	0.876	0.653	0.522
CTNT (pg/ml)	24.47±41.46	22.29±49.80	898.34±1633.08	34.97	0.992	<0.001	<0.001
Treatment							
Statin use (%)	6	26	45	44.437	<0.001	<0.001	<0.001
Aspirin use (%)	7	29	47	45.941	<0.001	<0.001	<0.001
Antihypertention therapy (%)	8	16	40	34.279	0.237	<0.001	<0.001

UA: unstable angina; SA: stable angina; BMI: body mass index; TC: total cholesterol; HDL: High-Density Lipoprotein; TG: Triglyceride; LDL: Low-Density Lipoprotein; hs-CRP: high sensitivity C-reactive protein; cTNT: Troponin T; TIMI: thrombolysis in myocardial infarction; The data shows as mean±SD.

### 3.2 Correlation between CXCL17, Gensini score and hs-CRP

**Figure 1:**
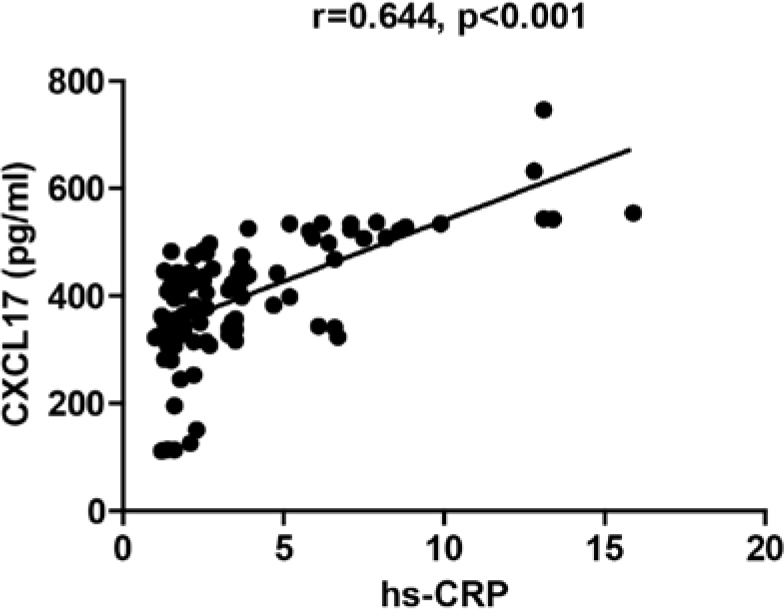
The correlation between CXCL17, hs-CRP. The CXCL17 and hs-CRP level in serum of patients with UA. UA: unstable angina.

**Figure 2:**
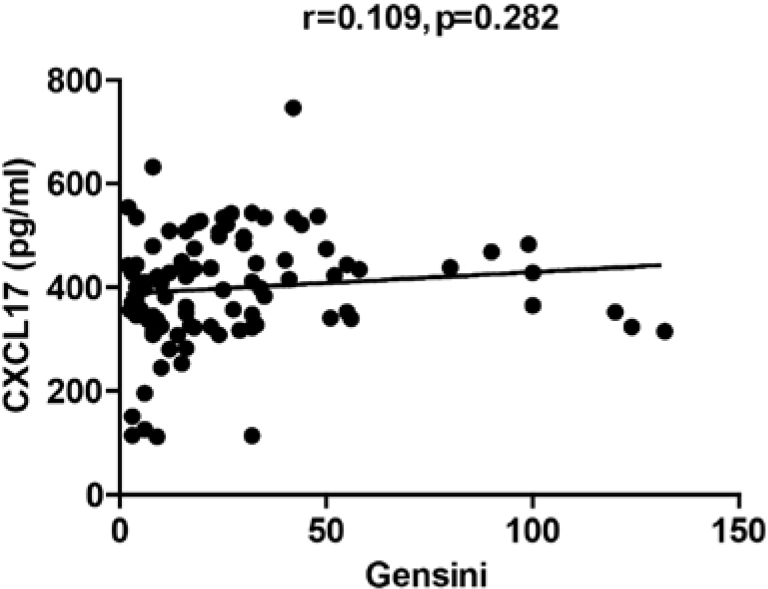
The correlation between CXCL17, Gensini score. The CXCL17 level in serum of patients with UA and the related Gensini score. UA: unstable angina.

Hs-CRP is a well-known traditional risk factor for CHD [8, 9], while Gensini score is a common indicator of the severity of CHD [10] Thus, we compared hs-CRP and Gensini score between UA and SA. The UA group had a significant higher level of hs-CRP (SA 1.98±0.70 vs UA 5.61±3.52, p<0.001) and Gensini score (SA 19.25±24.48 vs UA 35.26±29.48, P=0.004) as shown in Table 2. Subsequently, we compared whether CXCL17 was related to hs-CRP and Gensini score. According to Spearman’s correlation method, CXCL17 had a positive association with hs-CPR (Figure 1) (r=0.644, p<0.001), whereas no correlation was found with Gensini score (Figure 2) (r=0.109, p=0.282).

**Table 2:** Comparison of the serum hs-CRP and Gensini score between stable angina and unstable angina groups.

	SA	UA	t	P
Gensini score	19.25±24.48	35.26±29.48	-2.929	0.004
hs-CRP	1.98±0.70	5.61±3.52	-7.011	<0.001

UA: unstable angina; SA: stable angina; hs-CRP: high sensitivity C-reactive protein; The data shows as mean±SD.

### 3.3 Association between CXCL17 and the cardiovascular risk factors in unstable angina

In order to test whether CXCL17 was an independent risk factor for UA, a logistic regression analysis was made using different models (Model 1 and 2) as shown in Table 3. Adjustments for sex, age, body mass index, smoking, hypertension and diabetes were determined. After correction of the above related risk factors, the statistical analysis showed that CXCL17 was an independent risk factor for UA.

**Table 3:** Logistic regression analyses of association between serum CXCL17 levels and unstable angina.

CXCL17	β	S.E.	Wald	P	Exp(B)	95%C.I.for Exp(B)	
						Lower	Upper
Model1	0.018	0.004	22.375	<0.001	1.018	1.011	1.026
Model2	0.017	0.004	17.666	<0.001	1.017	1.009	1.025

Model 1: Unadjusted. Model 2: Adjusted for sex, age, BMI, smoking, hypertension, diabetes.

## 4 Discussion

Unlike acute myocardial infarction, there is no effective biomarker for unstable angina at present. Recent studies have found that many kinds of chemokines might be served as markers for unstable angina pectoris [11, 12]. For example, CCL2, CXCL8, CXCL9, CXCL10 and CCL7 are significantly up-regulated in patients with CHD [13, 14]. They are related to the severity of atherosclerosis independent of traditional cardiovascular risk factors [14]. CX3CR1, (CCL5/RANTES) and CC chemokine ligand-18 (CCL18/ PARC) are closely related to refractory unstable angina and can be used as a specific marker [11, 15], while CX3CL1 is used as a marker of response to statin therapy [16]. However, little is known about the biological function of CXCL17 in atherosclerosis which is one of the chemokine of CXC family. The previous studies have demonstrated that CXCL17 is abnormally expressed in multiple target tissues of oncology patients [17, 18]. Meanwhile, CXCL17 acts as a chemo- attractant for monocytes and macrophages, which suggested that it could play an important role in the angiogenesis of tumor development [6]. Moreover, CXCL17 expression could be induced in macrophages [19], especially in lung macrophages [20], and tightly co-regulated with vascular endothelial growth factor expression [6]. Although CXCL17 plays an important role in angiogenesis of tumors, it also has an anti-inflammatory effect in tissue repair [7]. A recent study exhibits that GPCR35 acted as the receptor of CXCL17 and is closely related to cardiovascular disease [3, 4]. In our study we showed that the serum levels of CXCL17 were an independent risk factor for UA, while there were no significant differences between patients with SA and the CG. The collected evidences suggested that CXCL17 could be used as a stable marker of UA, but not as a diagnostic tool for CHD.

Clinical studies have shown that the occurrence of cardiovascular events mainly depend on the stability of plaque [21], which is correlated to the severity of the inflammatory reactions [22]. Gensini score is commonly used to evaluate the severity of coronary lesions [10] without taking in consideration the stability of the plaque. In our study, Gensini score was significantly increased in patients with UA, but no correlation was found between the latter and CXCL17. This result indicated that CXCL17 had a possible role in the stability of the plaque and not into the degree of atherosclerosis.

Hs-CRP is an acute phase reactive protein, which is a classic inflammatory marker in AS [8, 9], especially in patients with acute coronary syndrome (ACS) [23]. Many acute phase proteins were found significantly increased in ACS patients, such as haptoglobin, alpha-1-antitrypsin, ceruloplasmin, alpha-1 glycoprotein. Their elevation is significantly correlated with CRP concentration, and appear to be closely related to the severity of coronary atherosclerosis and to myocardial damage [24]. CRP is showed to be significantly correlated to creatin kinase, lactate dehydrogenase, troponin I and with the incidence of major adverse cardiac events in ACS patients [25]. CRP levels are strongly related to myocardial damage more than to pre-existing inflammation [25]. High sensitivity C-reactive protein is used as a marker of unstable angina pectoris and acts as a prognostic factor for CHD [26, 27]. In our study, CXCL17 was positively correlated with the levels of hs-CRP. CXCL17 and hs-CRP were significantly higher in UA patients rather than in the SA group; this result can be explained by the increased inflammatory activity found in the unstable plaques [23, 28, 29]. CXCL17 levels were associated with inflammatory activity and with the instability of atherosclerotic plaques.

There are some limitations in this study. The sample size is small and larger cohort studies are necessary to confirm our findings. The interferences caused by cardiovascular drug treatment are not completely excluded. In the UA group, drugs (including statins, aspirin and anti-hypertension) were universally used compared to the other two groups, which could have some influence on inflammation. Nevertheless, the CXCL17 expression in UA group was still significantly up-regulated. Finally, considering that acute stress may have an effect on chemokines, we excluded from the study patients with acute myocardial infarction.

## 5 Conclusions

At present, little is known about the biological properties of CXCL17, which is mainly focused on tumors. Therefore, further research on the function of CXCL17 needs to be revealed. In our study, CXCL17 was found to be an independent risk factor for unstable angina. The serum levels of CXCL17 were significantly up-regulated in patients with unstable angina, while no significant differences were found between stable angina and the control group. Moreover, CXCL17 was statistically correlated with hs-CRP and was associated to inflammatory activity, as well as the instability of atherosclerotic plaques. Those results suggested that CXCL17 elevation may act as a potential biomarker to distinguish stable from unstable angina pectoris after exclusion of acute myocardial infarction.
